# Stereoscopic motion analysis in densely packed clusters: 3D analysis of the shimmering behaviour in Giant honey bees

**DOI:** 10.1186/1742-9994-8-3

**Published:** 2011-02-08

**Authors:** Gerald Kastberger, Michael Maurer, Frank Weihmann, Matthias Ruether, Thomas Hoetzl, Ilse Kranner, Horst Bischof

**Affiliations:** 1 Institute of Zoology, University Graz, Graz, Austria; 2 Institute for Computer Graphics and Vision, Graz University of Technology, Graz, Austria; 3 Royal Botanic Gardens Kew, Wakehurst Place, UK

## Abstract

**Background:**

The detailed interpretation of mass phenomena such as human escape panic or swarm behaviour in birds, fish and insects requires detailed analysis of the 3D movements of individual participants. Here, we describe the adaptation of a 3D stereoscopic imaging method to measure the positional coordinates of individual agents in densely packed clusters. The method was applied to study behavioural aspects of shimmering in Giant honeybees, a collective defence behaviour that deters predatory wasps by visual cues, whereby individual bees flip their abdomen upwards in a split second, producing Mexican wave-like patterns.

**Results:**

Stereoscopic imaging provided non-invasive, automated, simultaneous, *in-situ *3D measurements of hundreds of bees on the nest surface regarding their thoracic position and orientation of the body length axis. *Segmentation *was the basis for the *stereo matching*, which defined correspondences of individual bees in pairs of stereo images. Stereo-matched "agent bees" were re-identified in subsequent frames by the *tracking *procedure and *triangulated *into real-world coordinates. These algorithms were required to calculate the three spatial motion components (dx: horizontal, dy: vertical and dz: towards and from the comb) of individual bees over time.

**Conclusions:**

The method enables the assessment of the 3D positions of individual Giant honeybees, which is not possible with single-view cameras. The method can be applied to distinguish at the individual bee level active movements of the thoraces produced by abdominal flipping from passive motions generated by the moving bee curtain. The data provide evidence that the z-deflections of thoraces are potential cues for colony-intrinsic communication. The method helps to understand the phenomenon of collective decision-making through mechanoceptive synchronization and to associate shimmering with the principles of wave propagation. With further, minor modifications, the method could be used to study aspects of other mass phenomena that involve active and passive movements of individual agents in densely packed clusters.

## Background

**Figure 1:**
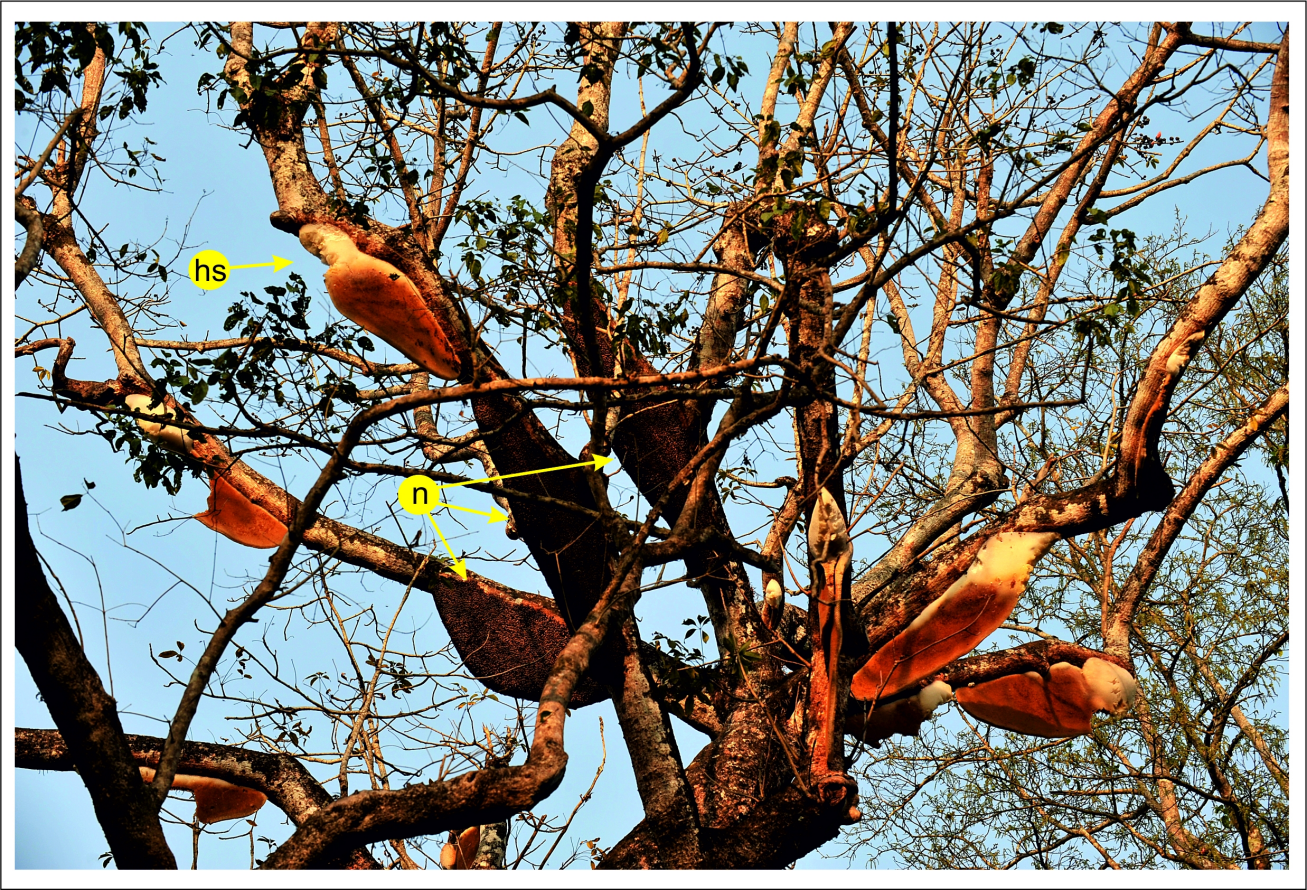
**Typical colony aggregation of Giant honeybees in the natural habitat**. The investigated colony aggregation in Chitwan National Park, Nepal, comprised 10 nests. The photo was taken in February 2009. One week earlier all but three colonies (*n*) had absconded, leaving their combs behind. Honey buzzards had destroyed some of the honey stores (*hs*) while consuming the brood.

**Figure 2:**
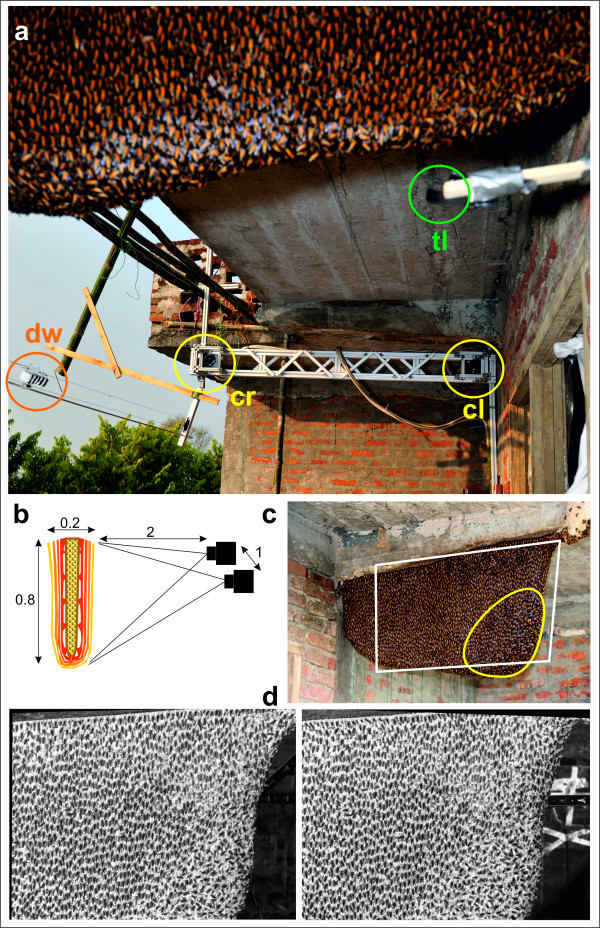
**Acquisition of stereo images**. *(a) Pair of synchronized *cameras (c_r_, right camera; c_l_, left camera, yellow circles) with a resolution of 2352 × 1728 pixels at 60 Hz recording stereo images of a Giant honeybee nest on a balcony of a house in Chitwan, Nepal. Before the measurements, the cameras were fixed to an aluminium carrier rig and calibrated; the orange circle refers the movable dummy wasp (dw; note the black and white stripes) on a cable-car device; tl, trigger light (green circle) for the synchronization of the HD cameras and additional sensory equipment (cf see Additional File 1, Movie S1). The frame grabbers and the computer were located in the adjacent room to the right side. (b) Schematic showing the camera setup at a Giant honeybee nest with a central comb with several layers of worker bees (termed "bee curtain"); numbers indicate distances in metres. (c) HD image of the experimental giant honeybee nest. The white rectangle indicates the area recorded by stereo imaging. The yellow line marks the "mouth zone", which is the most active zone of the nest where bees depart and arrive. (d) Example of a pair (left and right) of stereo images as acquired by the stereo cameras.

**Figure 3:**
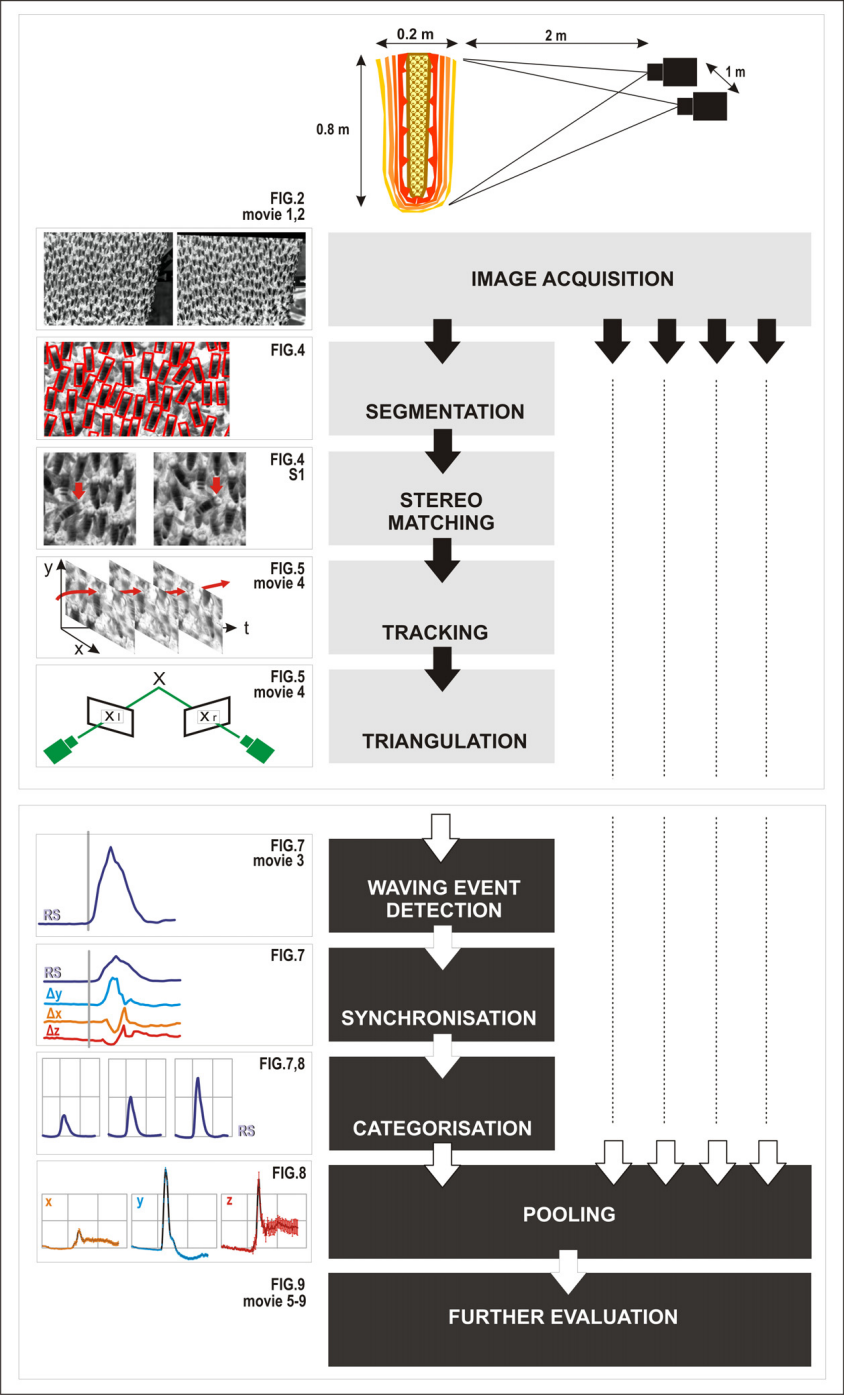
**Schematic of the processes of 3D stereoscopic imaging and its application to the analysis of shimmering waves in Giant honeybees**. From top to bottom: The experimental nest was captured by two frame-locked video cameras positioned at an angle of 30° two metres in front of the nest. In the offline data assessment phase, the acquired images were processed as follows: *Segmentation *distinguished single agent bees in the densely packed clusters of bees on the surface of the nest; *stereo matching *identified corresponding agent bees in both paired images. These two processes enabled *stereo tracking *of the agents in subsequent frames throughout whole film scenes and *triangulation *of their thoracic positions regarding the three dimensions of space (x,y,z). Following the stereoscopic process, the images are evaluated. For example, the arrival of the wave front at an individual agent can be recognized by a movement detection algorithm also producing a measure for the response strength *RS *of the agent. This allows synchronization and pooling of 3D data (Δx, Δy, Δz) of wave "episodes" (for definition, see text) of individual agent bees at different positions of the nest for a variety of behavioural applications. The bright-grey flow charts address the stereoscopic single-agent analysis from *segmentation *to *categorization*, the dark-grey flow charts refer to further processing such as synchronization and pooling of single-agent data. The arrows and dashed lines at the right symbolize that the stereoscopic process produced further data for hundreds of agent bees simultaneously. The panels to the left of the flow charts illustrate the results of the processes shown to their right (see referenced figures and movies for details).

Giant honeybee (*Apis dorsata*) nests [1-7] constitute a matrix of densely clustered individuals arranged in a multi-layered stratum, the "bee curtain" [8], around a central, flat comb (Figures 1 and 2). Collective behaviours such as mass flight activity and colony defence [7,9] are affected by the functional principles of the 3D architecture of this bee curtain. Defence strategies against predatory wasps [10,11] include shimmering behaviour [3,12,13] (see Additional File 1, Movie S1), the ultimate evolutionary goals of which are not fully understood. Proximate aspects such as the underlying logistic principles of wave generation and propagation are also unclear [13]. Shimmering waves are produced by individual surface bees that consecutively flip their abdomens upwards, typically at an angle of 90° [14] within 100 ms. Information may be transmitted by "bucket-bridging" from one surface bee to an adjacent one, similar to Mexican waves in football stadiums [15], where information is also transferred by repetitive movements of individual participants consecutively. Shimmering waves can also "jump" from one excited group of surface bees to another [13], which can co-occur with bucket-bridging. Ultimately, shimmering may not only provide visual patterns for external addressees such as predatory wasps, but may also play roles in colony-intrinsic communication [13,16]. We hypothesized [13,16,17] that shimmering surface bees affect sub-surface layers in the bee curtain, providing mechanoceptive signals for the colony members in all curtain layers, including those that do not actively participate in a wave. We studied shimmering in Giant honeybees under field conditions (Figure 1 and 2) in Chitwan (Nepal), with the goal to simultaneously measure the motions of hundreds of surface bees in the three directions of space to obtain a detailed view of the movements of individual bees within the entirety of the bee curtain. This was achieved by an adaptation of the stereoscopic imaging principle [18] with its fundamental algorithms (Figure 3): *segmentation *[19], *matching *[20,21] and *reconstruction *[22,23] by *tracking *and *triangulation*.

Challenges arose, firstly, from the requirement to individually track identified surface bees, hereafter termed "agent bees", in successive frames (*ff*) of a shimmering process, which is difficult because all agent bees are extremely similar in morphology, are densely clustered, and show rapid movements in 3D during their abdominal flipping. Secondly, an individual bee sensing an incoming wave front due to the movements of her neighbours is free to decide whether or not to participate, and if so, whether to participate strongly or weakly. It is critical to distinguish active "movements", i.e. abdominal flipping, from passive "motions" caused by the surrounding bee curtain. Individuals that participate weakly in shimmering are difficult to detect by automated analysis. Thirdly, data of the positional coordinates of agents are, for the external observer, stochastic and noisy. This is because collective behaviours in eusocial insects are determined by self-organization [13,24-26], whereby patterns at a global level of a system emerge from numerous interactions among the lower-level components of the system, and rules specifying interactions among the systems' components are executed using only local information, without reference to the global pattern [24]. Hence, the data describing the behaviour of the lower-level components (here: the agent bees) appear stochastic and noisy, although the global effect (here: shimmering) is clearly recognisable. Fourthly, high-resolution and high-speed cameras are essential to produce rich data sets with high geometrical resolution, detecting movements of single agents within fractions of a millimetre across an entire nest that can span up to 1.5 m in diameter. The equipment must deliver reliable, accurate data, even under harsh field conditions in the natural habitats of Giant honeybees where electronic equipment may fail due to high temperature, air humidity and solar irradiance. Lastly, Giant honeybees are among the most aggressive insects known [8]. To avoid unwanted colony arousal, data recording must be non-invasive, keeping a distance of at least 2 m between equipment and nest.

We propose a method for the automated identification of individual bees within the densely packed clusters of the bee curtain surface, including the computation of the 3D locations of individual agent bees, following them over time and identifying the arrival of shimmering waves at those agents. The data obtained with this method can enhance our understanding of the generation and propagation of shimmering waves in Giant honeybees and of the contribution of nest members to intra-colonial communication.

## Results

**Figure 4:**
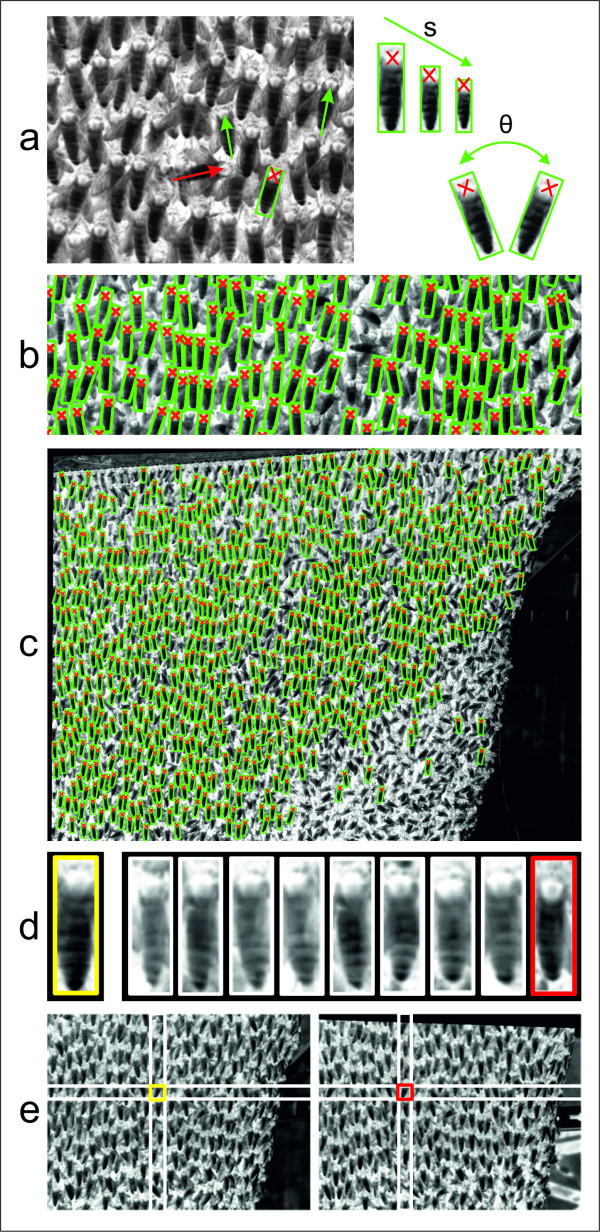
**Segmentation and stereo matching of agent bees**. A Giant honeybee nest represents a compact matrix of individuals, arranged in multiple layers where bees adhere to subjacent layers with their legs. The surface layer and parts of the subsurface layer are visible in a-c and e. The luminance values have been inverted for better contrast. (a) Template patterns representing typical bees of varying size (s) were rotated at discrete angular steps (-11.5° ≥ θ ≥ + 11.5° in 2.86° steps). Outlier agent bees that did not match any of the prototypes (e.g. in turbulence areas such as the *mouth *zone [8] or convection holes; indicated by the red arrow in a) were not segmented. Segmentation resulted in assigning a rectangular area (green rectangles in a-c) to each individual bee within which the thoraces were defined (red crosses in a-c). (b,c) Data plots of a NCC (Normalized cross-correlation) segmentation of 505 identified agent bees: images in a and b are details from c, using five templates with a variation in orientation of 23°, and a maximum scale variation of 13.7% corresponding to 9 *px *at a reference bee length of 65.63 ± 0.45 *px*; n = 70. (d) Rectified honeybee templates demonstrating the challenges of stereo matching (see text): the segmented agent bee (yellow line in d,e) recorded by the left camera is to be identified in the paired image produced by the right camera (red line in d,e) only by the NCC similarity criterion. (e) Pair of rectified stereo images, in which all epipolar lines were aligned horizontally, so that corresponding points lie in the same image row.

**Figure 5:**
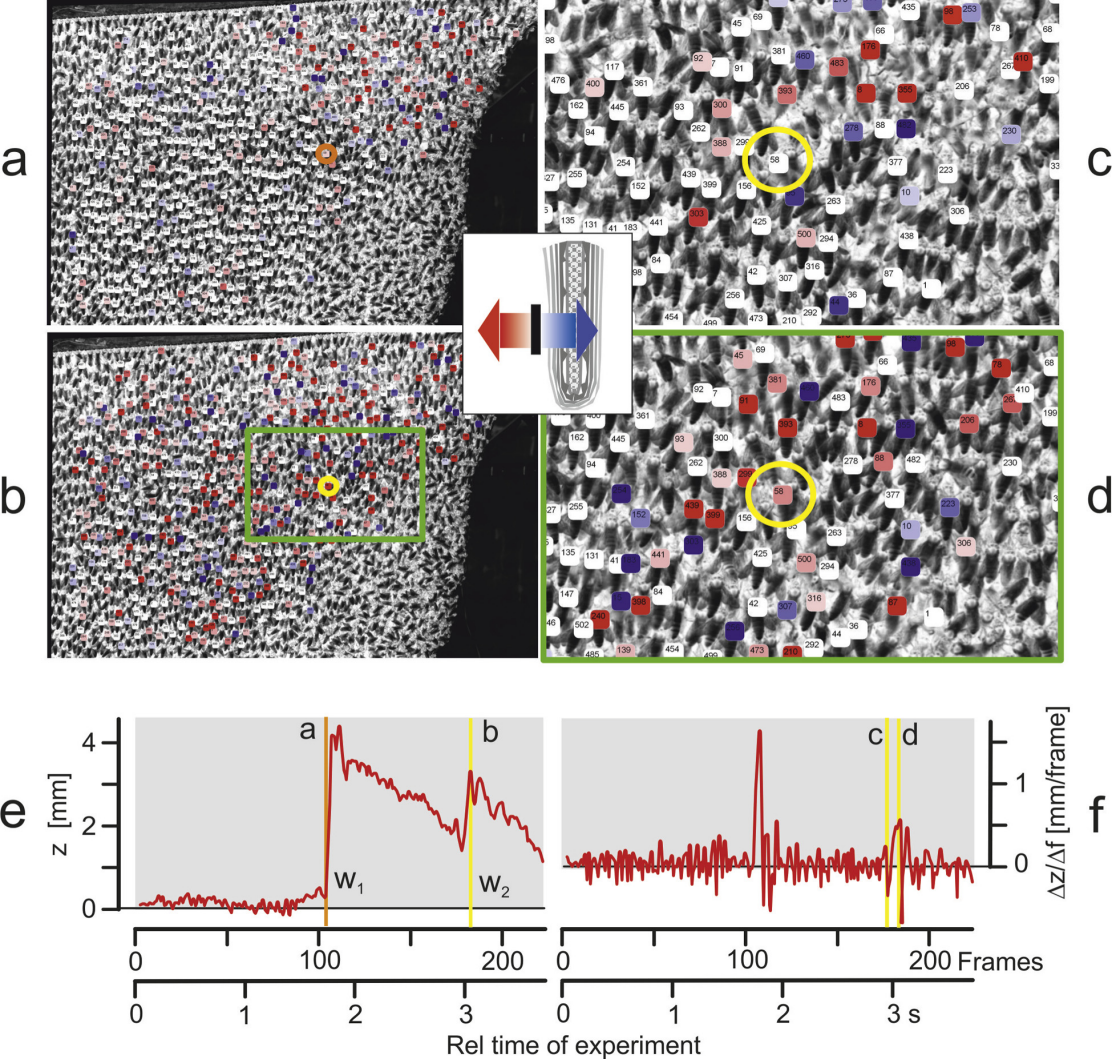
**Triangulation and tracking of agent bees**. Sample images (left camera) illustrate the triangulation and tracking challenges; (a) and (b) show the frames *f *102 and *f *182, respectively; (c) and (d) show details from *f *177 and *f *184, respectively. In a-b and c-d, luminance values have been inverted for better contrasting of the abdomens. Agent bees identified and triangulated into real-world coordinates were colour-coded regarding their z-positions and assigned unique numbers. The z-values and their differentials (Δz/Δf; fps = 60 Hz) were categorized in 2 × 14 steps of strength (see inset), towards the comb (blue shadings), and away from the comb (red shadings). Agent 58 is indicated by an orange circle in a, and by yellow circles in b-d. (e,f) Time courses of the z-deflection of agent 58 in mm (e) and its differential in mm/frame (f); the experiment was started at time zero; therefore the z-position of agent 58 prior to wave w_1 _(in e) was not affected by any preceding wave process; vertical bars refer to the time points of the samples (a-d) and have the same colours as the labels of agent 58 used above.

To automate the analysis of roughly 500 agents in a series of about 900 images per video sequence, we designed and implemented an algorithm using stereo videos as the input to calculate a three-dimensional movement path for each bee. In the following, we first describe in the Technical section (Figures 2 and 3) the implementation of image acquisition and of the chain of algorithms by which agent bees were identified and tracked. In each pair of stereo images, hundreds of surface bees were identified regarding their position and orientation in the images (Figure 4a-c). This *segmentation *was the basis for the *stereo matching *(Figure 4d-e and 5), which defines correspondences of individuals in pairs of stereo images. Stereo-matched agents were re-identified in subsequent frames by the tracking procedure and triangulated into real-world coordinates regarding their 3D locations and orientations (see Additional Files 2, 3 and 4, Movies S2, S3 and S4). In parallel, the arrival of shimmering waves at the selected agents was automatically detected within a path of stereo images. "Wave events" that triggered a response of an individual agent (to flip the abdomen or stay quiescent) were used as a means for synchronizing wave movement paths of hundreds of agents to achieve appropriate statistical evaluation of the data. In the Application section of this paper, we exemplified the use of the method to achieve a deeper understanding of selected behavioural aspects and to develop a model that describes the physical principles of abdominal flipping.

### Technical Section: Algorithm and Implementation

#### Image acquisition

At the experimental nest, the individual and collective motions of surface bees were captured in a stereoscopic video sequence (Figure 2). Pairs of synchronous, frame-locked images recorded from different viewpoints (" stereo images") were obtained by two digital cameras (for technical specifications, see Additional File 5, Table S1) placed symmetrically ~2 m in front of the nest with a baseline of 1 m between the cameras. The cameras were mounted on a solid carrier and connected to a computer. To elicit shimmering waves, a striped dummy wasp made from Styrofoam was fastened with fine yarn to a cable-car device for computer-controlled movements at variable velocity (0.1-0.5 m/s) and direction (Figure 2). In the experiments described here, the dummy wasp was moved at an angle of 90° to the nest surface (shown on the right side of the experimental nest in the images, see Additional File 1, Movie S1 and Additional File 6, Movie S5).

The stereo cameras had a resolution of 2352 × 1728 pixel (*px*). From the given working distance of 2 m, and with a calibrated focal length of 53 mm, about two thirds (700 mm in diameter) of the nest were recorded, whereby one *px *represented ~0.30 mm in metric real-world coordinates. Therefore, a bee with an abdomen width of 6 mm was captured by an image region of roughly 20 *px*. The cameras were able to capture 60 frames per sec (*fps*) and so resolve the abdomen-flipping phase of an individual bee of 200 ms within 12 frames (*ff*). The resulting data stream of 480 MB/s was buffered to RAM and subsequently stored to disk. Consequently, the total of 8 GB of existing RAM allowed an acquisition time not longer than 15 s. The cameras were connected as master and slave; one camera generated a trigger signal for the second camera to achieve temporal synchronization. The cameras were mounted on a carrier rig (Figure 2), which enabled their positioning at variable baselines *b *(typically 1 m) with a variable stereo angle α. The baseline was calculated from the required working distance of d = 2 m and a stereo angle of α = 30° by

(1) $$b=2\;d\tan\left(\frac{\alpha }{2}\right)$$

The expected depth error *e_z _*was estimated on the assumption of orthogonal cameras according to

(2) $$e_{z}=\frac{1}{\sin\alpha }m\;e_{img}\cos\frac{\alpha }{2}$$

with *e_img _*as the pixel error of stereo matching; *m*, the magnification at the given working distance. Under the given conditions (α = 30°, *m *= 0.3 mm/*px*; e_img _= 1 *px; *equation 2) a resulting depth error of *e_z _= *0.6 mm was achieved. In summary, stereo images were typically recorded over 15 s, capturing 900 *ff *per camera at a rate of 60 Hz and at a spatial resolution of 0.3 mm per *px *within a measurement volume of 700 × 500 × 150 mm^3 ^(x,y,z) whereby the nest-specific axes were defined as *x *(= horizontal: left-right), *y *(= vertical: up-down) and *z *(= directions towards and away from the comb).

#### Segmentation

Agents were segmented by comparison with pre-generated template images (Figure 4a). Normalized cross-correlation (NCC) [27] was used to define similarity. The correlation of an image *f*_*m *× *n *_with a smaller two-dimensional template pattern *t*_*k *× *l*_, which shows a sample bee, was assessed according to

(3) $$c\left(u,v\right)=\sum_{\text{x,y}}f\left(x,y\right)\;t\left(x\text{-}u,y\text{-}v\right)$$

To calculate the correlation for a single pixel location in *f *with coordinates (*u,v*), the template *t *was overlaid to the region around this pixel and the sum of products of overlaying pixels within this region was calculated. In equation 3 *x *and *y *denote the coordinates of all overlaying pixels in this region. Normalized cross correlation extends classical correlation and is robust to variations in illumination, and is formulated by normalizing the image and the template vectors to the unit length of the image according to

(4a) $$\gamma \text{1}\left(u,v\right)=\left[f\left(x,y\right)\text{-}\;{\bar{f}}_{u,v}\right]$$

(4b) $$\gamma \text{2}\left(u,v\right)=\left[\text{t}\left(x\text{-}u,y\text{-}v\right)\text{-}\bar{t}\right]$$

(4c) $$\gamma \left(u,v\right)=\frac{\sum_{\text{x,y}}\gamma \text{1}\left(u,v\right)\gamma \text{2}\left(u,v\right)}{\sqrt{\sum_{x,y}\gamma \text{1}{\left(u,v\right)}^{2}\sum_{x,y}\gamma \text{2}{\left(u,v\right)}^{2}}}$$

Here, $\bar{t}$ is the mean of *t*, and ${\bar{f}}_{u,v}$ is the mean of *f *in the template.

The goal was to identify individual bees at the surface of a densely structured matrix in a multi-layered nest (Figures 1 and 2). For that, template patterns were defined from representative agent bees with varying orientations and scales (Figure 4a); such templates included the abdomen and the thorax rather than the head which is often concealed by other surface bees. Each template type was matched against every position (*u,v*) in the image. Local similarity maxima, which were obtained by non-maximum suppression, represented successful matches. To compensate for overlaps, weak local maxima in the vicinity of 20 *px *around a more dominant one were eliminated. Consecutively, the segmentation routine was repeated with scaled and rotated templates to account for variations between the individual prototypes in alignment and size. The charts (Figure 4b,c) exemplify template matching using five templates with a variation in orientation of 23° (which represented a typical measure of the maximum deviation angle in the *quiescent *[7] areas of the nest), and with a scale tolerance of 14% (corresponding to 9 *px*). The procedure recognized hundreds of surface bees in the bee curtain (Figure 2c and 4c) under *quiescent *[7] conditions when they were inside the orientation limits and were not overlapped by other bees.

#### Stereo matching

*Stereo matching *allowed us to compare the template region of an agent in the left image with the paired, frame-synchronized right image, identifying the best match for the correct correspondence. This process could have followed the same strategy as that in *segmentation*. However, the high degree in pattern similarity of neighbours and the differences in perspective made it difficult to assign unique correspondences. Figure 4d,e illustrates the challenges arising in *stereo matching *exemplifying rectified honeybee templates: the agent segmented in the left stereo image coded by a yellow frame was identified in the paired right images by the similarity criterion and selected out of nine candidates; finally, the *template matching *was successful for the agent bee in the red rectangle.

From the segmentation algorithm, the thorax positions of N individuals in the left image and M individuals in the right image were known. This is given by

(5a) $$S_{left}=\left\{t_{p}^{left},p=1..N\right\}$$

(5b) $$S_{right}=\left\{t_{i}^{right},i=1..M\right\}$$

To calculate the three-dimensional locations of segmented thoraces, we first identified a set of correspondences *c_n _*= (*p*, *i*), which followed the rule that $t_{p}^{left}$ and $t_{i}^{right}$ were images of the same thorax. One such correspondence is marked in Figure 4e. It is important to note that both stereo images were rectified [22], with the consequence that correspondences can only lie in the same row of both images. However, assessing the correspondence between agent bees was still challenging because of the presence of patterns with repetitive similarity. The method we used to solve this problem as a discrete energy minimization task is described in detail by

(6a) $$\arg_{d}\min E\left(d\right)$$

(6b) $$E\left(d\right)=E_{data}\left(d\right)+\lambda E_{smooth}\left(d\right)$$

and in Additional File 7, Text S1 and Additional File 8, Figure S1 [28,29].

#### Tracking

**Figure 6:**
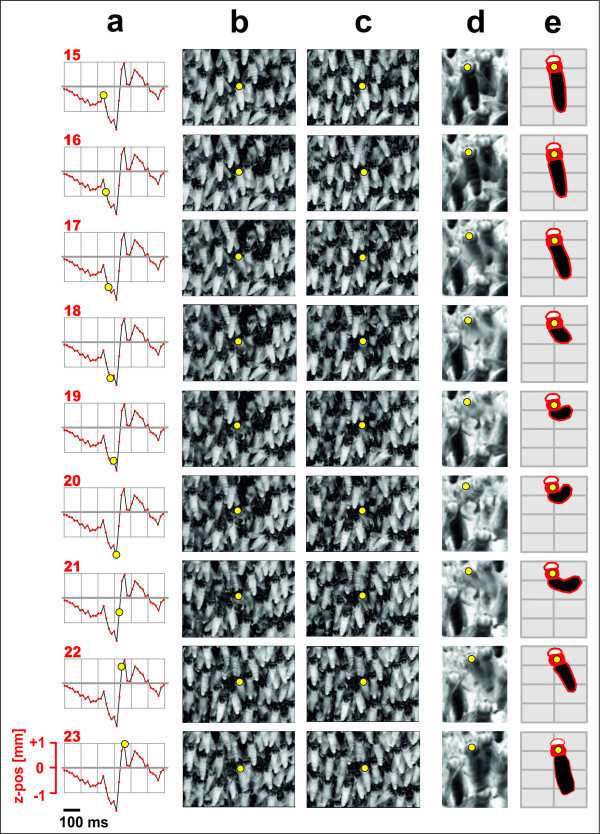
**Changes of the z-position of the thorax of agent 58 during abdominal flipping**. (a) the z-value during a shimmering episode (the same as displayed in Figure 6, w_2_), computed by stereo tracking; the grid ranges from +1 (away from the comb) to -1 mm (towards the comb) (ordinate), and refers to 600 ms (abscissa, see horizontal bar); the rows refer to nine successive frames starting with frame 15 (frame 0, not displayed here, refers to the first in a sequence of 40 images which featured the respective wave event at agent 58, see Figure 6); (b,c) the pairs of left and right stereo images; (d) detailed cut of agent 58 from the left camera image (inverted luminance); (e) contour display of agent 58 extracted from d, with abdomen (black), thorax (red) and head (white). The yellow full circles mark the z-value of the thorax in (a) and the thorax position in (b-e). Note that the abdominal flipping started already when the thorax of the agent moved towards the comb, represented by the negative transient in (a). Within 100 ms the abdomen was thrown upwards at more than 90°, which can be seen in the respective images in the projection of the abdomen with the shortening and curving effect (d,e). From frame 20 onwards the abdominal flipping declined and the thorax was pushed by a strong force away from the comb (a).

The thoraces of agents were *stereo tracked *throughout the stereo sequence to obtain their 3D motions (Figure 5 and 6, see Additional Files 2, 3 and 4, Movies S2, S3 and S4). The *tracking *method was closely related to the *segmentation *process; it defined a 20 × 20 *px *template around the thorax of a segmented agent at time *t *and compared it with a larger search region of 40 × 40 *px *in the subsequent image at time *t+*1. The location with the maximum NCC value was selected as an agent's new position. Here, the rapid, forceful abdominal thrusts of shimmering agent bees (Figure 6; see Additional Files 9, 10 and 11, Movies S6, S7 and S8) hampered the tracking success and required a higher robustness of the matching procedure. Utilizing a symmetry criterion deduced from the time course of the abdominal movements of each agent (Figure 6d-e), the search region in the current frame was compared with a set of 20 × 20 *px *templates from the preceding 15 frames. Consequently, the templates produced in the upward thrusting phase were successively compared with those from the downward phase. The tracking results obtained from the left and right images were finally fused by projecting them to their mean image row. This double matching produced accurate results avoiding drifts of the tracking points during abdominal flipping.

Despite the introduction of the symmetry criterion in the tracking procedure up to 16% of the 400-600 identified agents were "lost" in the course of multiple waves. Nevertheless, we were able to compensate for these losses by restarting segmentation and stereo matching after each wave to retain stable numbers of agents throughout the evaluation path. We also minimized standard errors by the offset-correction of the positional coordinates of each agent. The arithmetic mean of the first six of the initial 30 frames prior to the arrival of a wave was subtracted from all data in a 90-frames episode (1500 ms). Offset correction also compensated for the residual spatial deflections with a time constant of 2-3 s which was characteristic for the descending process after a shimmering wave process caused by the receding motion of the bee curtain exemplified in Figure 5a-b and in the Additional File 3. Movie S3 shows the original data before offset-correction and prove that 3D stereoscopic imaging delivers the positional data without high-pass filter effects.

#### Triangulation

From each known correspondence *c_n _*= (*p*,*i*), the three-dimensional thorax location *T_n _*was calculated by triangulation [22]. The known thorax locations in the image space and $t_{p}^{left}$ and $t_{i}^{right}$, in combination with known camera projection matrices *P^left ^*and *P^right^*, were used to formulate the linear problem in

(7) $$\left[\begin{array}{cc} {\left[t_{p}^{left}\right]}_{\times } & P^{left}\\ {\left[t_{i}^{right}\right]}_{\times } & P^{right} \end{array}\right]T_{n}=0$$

The term [*h*]_× _here denotes the 3 × 3 cross matrix of a three-dimensional vector *h *(see [22]). A least-squares solution to the homogeneous equation system was obtained by solving the linear equation system. To maintain accuracy under the given field conditions, the stereo imaging system was calibrated daily using a standard calibration method [30] to maintain geometric consistency.

#### Detecting the arrival of a wave at individual agents

**Figure 7:**
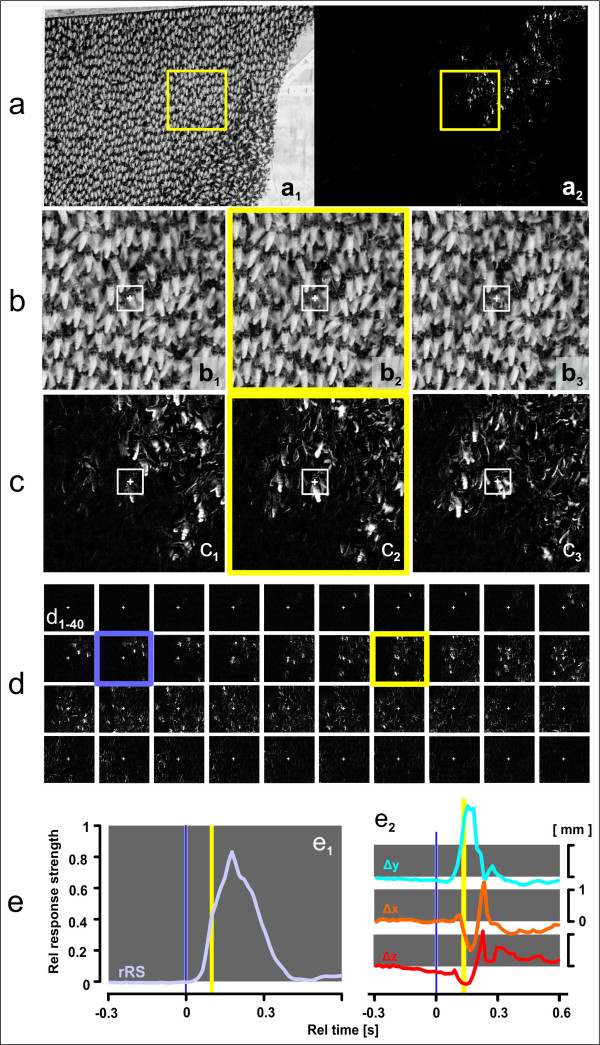
**Detection of the wave front**. (a) Frame ("*f*") 177 (of 900, taken by the left camera within 15 s at fps = 60 Hz) recorded 6 frames after the arrival of a wave at agent 58 (compare vertical yellow lines in e); (a_1_) original image; (a_2_) differential image calculated by pixel-operated subtraction; the white spots show changes in luminance from *f *176 to *f *177. Yellow rectangles show 500 × 500 pixel areas around agent 58 for the frame 177. (b_1-3_,c_1-3_) Details of *ff *176-178 (images from left to right) as original and differential images; white rectangles (15 × 15 mm) denote the sensor regions of interest (sROIs) for measuring the motions of (and around) agent 58 (labelled with a white cross). (d_1-40_) Survey of the differential images, exemplified in (c_1-3_), but for 40 frames; the arrival of the wave (t_0 _= 0 s; violet vertical bars in e) was calculated for *f *171 (violet rectangles in d) and identified as the time at one frame before agent 58 was affected by the incoming wave. (e_1_) Relative response strength (*rRS*), expressed relative to the maximum *RS *value found in 605 agents. (e_2_) Relative (offset-corrected, see text) changes of the positions of the thorax of agent 58 in the course of shimmering in mm: orange, horizontal directions (Δx: positive direction is to the right side); blue, vertical directions (Δy: positive direction is upwards); red, directions towards and from the comb (Δz: positive direction is away from the comb).

**Figure 8:**
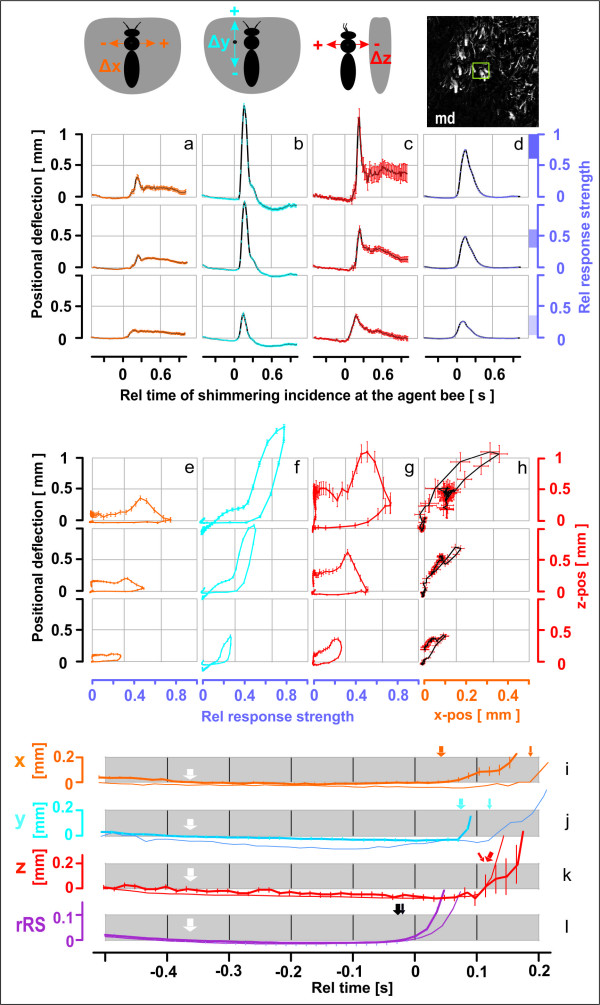
**Positional changes of selected agent bees on arrival of the wave front**. (a-d) Time courses of positional coordinates of the thoraces of 605 agent bees during two shimmering waves (900 frames). Time zero is defined by the arrival time of the wave at an individual agent; black curves represent arithmetic means; vertical bars, s.e.m.; colours show x-deflection (orange), y-deflection (blue), z-deflection (red) and *rRS*-values (violet); the positional (Δx, Δy, Δz) directions and the assessment of the *rRS *value by movement detection (md, see Figure 7) are defined in the schematics on the top. The waves were sorted regarding ten *RS*-levels, which consecutively were arranged in three groups, "high" (a-d, e-h; upper rows of charts), "middle" (middle rows) and "low" (bottom rows); the respective areas of *rRS *are shown on the right ordinate of a-d by the full rectangles of three different shadings of violet (n_high _= 725; n_middle _= 2571; n_low _= 1471 episodes); see Figure 9a for the detailed distribution of the *rRS *values. (e-g, see black-coloured ordinates on the left), correlations between the positional coordinates of agent bees (a-c) and *rRS *(d) over time. (h, see red-coloured ordinates on the right), correlations between the Δx- (a) and Δz-coordinates (c) over time, the arithmetic means are black in (h). (i-l), time courses of the positional components and *rRS *for the middle (means only, thin curves) and high (means ± s.e.m., thicker curves) range; amplified time scale; the arrival of the wave is marked by thick (high *rRS *range) and thin (middle *rRS *range) arrows. Note that the curves start in the pre-trigger period with slightly negative trends (white arrows at the left), 0.3-0.4 s before the waves arrived (black arrows in the *rRS *curves) at the agent bees.

For the diagnosis of the behaviours of agent bees it is essential to distinguish *active movements *(caused by actively flipping the abdomen) from *passive motions *(caused by the motions of the bee curtain in the surrounding of an agent). For that, the precise time of the arrival of a wave at the agent's position must be determined. A reliable trigger criterion was found by detecting the luminance changes in two sequential frames (*f*_i-1_,*f*_i_) in a pixel-wise subtraction creating a *difference image *(Figure 7 and 8d, see Additional File 12, Movie S9). We defined a *sensor region of interest *(sROI*_a_*) of 60 × 60 *px*, corresponding to 18 × 18 mm in real-world coordinates, around each agent's thorax and recorded the mean difference in sROI*_a _*luminance by Δ*L_a _*= Δ*lum *(*f*_i-1_,*f*_i_), out of 3600 *px*. The size of sROI*_a _*was chosen in conformance with the mean side-to-side distances between surface bees (12.67 ± 1.79 mm; mean ± ME, n = 50). Δ*L_a _*was used to quantify the effect of an arriving wave at an individual agent; the noise level of Δ*L_a _*≤ 5 (corresponding to "black" in the *difference image*) represented the state "motionless" and Δ*L_a _*= 255 ("white") "maximum movement". The arrival of a wave at an agent was defined two frames before the Δ*L_a _*values had exceeded the threshold luminance value Δ*L_th _*(Δ*L_a _*> Δ*L_th _*with Δ*L_th _*= 10) within three successive frames (Figure 7 and 8d). Typically, Δ*L_a _*peaked within 100 ms, and the maximal values defined the "response strength" (*RS*). For each agent, we considered thirty frames (500 ms) before and sixty frames (1000 ms) after time zero of the arrival of the wave and linked the *RS *values with the positional 3D data. Detection of incoming waves was compromised if the distances between neighbouring bees were smaller than 10 mm, whereby inactive agents could be erroneously classified as active in close vicinity with active neighbours. If required, correction is possible by reducing the area of sROI*_a_*. Here, we chose not to do so, because such compromising cases were rare and the errors were compensated for by the wealth of data recorded (Figure 8).

### Application Section: Behavioural Studies

To demonstrate the applicability of the method we focused on selected behavioural aspects regarding shimmering waves. Here, data of only a single spreading direction were analysed for waves seemingly spreading from the right side to the left side of the experimental nest (n_waves _= 14; n_episodes _= 4672; n_agents _= 605; when a shimmering wave hits an individual agent bee, a shimmering "episode" can be defined which includes the active and passive movements of this agent bee shortly before, during and after the arrival of the wave front). For all these behavioural examples, the statistical proof is based on synchronizing shimmering episodes by detecting the arrival of the wave at the immediate neighbourhood of the agent bees and on stereo tracking thoracic positions. In the first subchapter the primary motion components of selected agent bees are identified (Figure 5, 6, 7 and 8, see Additional File 2, Movie S2) which could be potential candidates to enforce the mechanical process of wave generation [31-33]. Second, for the same agents their (passively induced) motions were traced hundreds of milliseconds before the arrival of a wave, that is when the decision has to be made if and when to participate (Figure 8i-l) in a wave. Finally, we classified the modes of participation on the single agent level (Figure 6, 7 and 8d).

#### Identifying primary motion components of agent bees during shimmering

A shimmering wave arriving at an agent provokes characteristic positional changes (Figures 5, 6, 7 and 8, see Additional File 2, Movie S2). Regarding the y-components, 70 ms after wave arrival, as defined by the detection method described above (black arrows in Figure 8l), a strong upward movement occurred (blue arrows in Figure 8j); 20 ms later, the agent bee started to move in the z-direction, first she shifted slightly negatively, i.e. towards the comb, then strongly away from the comb (red arrows in Figure 8k); this biphasic z-motion is displayed more explicitly in single-agent data (cf. Figures 6 and 7e_2_). It is important to differentiate between the weak, positive transient deflections which resulted from the oncoming wave due to the general swinging of the bee curtain in the vicinity of the agent bee, and a strong, positive z-deflection which indicated "active" participation in the wave through abdominal flipping. With higher response strengths (*RS *values) the positive y-deflections started earlier (Figure 8j) and the positive z-motions later (Figure 8k) due to a more pronounced preceding negative phase of the z-component (cf. Figure 6). The horizontal components were the weakest of all, and the data illustrate that the positive x-components corresponded strongly with the positive z-components (r = 0.39; P < 0.001; n = 4782 wave episodes at agent bees; Pearson Product Moment Correlation Test); this happened at lower *RS *values at a lower intensity and with increasing time lags (Figure 8h,i,k). The deflections regarding x-, y- and z-directions seem to be very complex because they are caused by the specific attributes of the functional architecture of the bee curtain regarding an agents orientation (head up and abdomen downwards) and the coupling (physical contact) between the colony members of the different layers [8,13,16,17]. More details are given in the discussion part.

#### Motion detection at an agent bee prior to the arrival of a wave

To produce the typical cascadic shimmering waves that are visually recognized by external addressees [16], each surface bee needs to respond to an oncoming wave within a time window of less than 100 ms. For that, she has to decide whether or not to flip the abdomen, and if she does, she can raise her abdomen with gradual strength. Her decision is particularly linked to mechanical cues sensed tens of milliseconds prior to the arrival of the wave front. The possibility that movements of her immediate neighbours are also perceived visually cannot be excluded, but it would probably take too long to synchronize her abdominal flipping with the wave front only by visual input; the duration of senso-neural processes associated with image perception may last more than 150 ms [34] to prompt behavioural decisions [13,16,35]. However, the visual domain is important, in particular to perceive threatening stimuli such as predatory wasps hovering around the nest. Such visual patterns are key to initialize shimmering [13,16] and to collectively drive its spreading direction [35]. Mechanical cues, although less understood [13,16,35], must be essential for the wave spreading process. Surface bees are well endowed to sense mechanical cues, as they cling on to bees of the subsurface layer with their six legs, acting as a potent web of mechanoreceptors. In this paper, 3D stereoscopic imaging provided evidence for mechanoceptive cues, which were traced at individual agents in terms of small increments up to 300 ms before the wave front arrived (Figure 5e and 8i-k). Statistically proven for the investigated scenario of waves with high *RS *values (see Figure 7a for the z-component, test not shown here) spreading from the right to the left side, a wave typically pressed the agent bees slightly (< 0.2 mm) towards the nest, then shifted them into the direction of the oncoming wave, and lastly slightly downwards. However, recordings of agent 58 (Figure 5 and 6) illustrate in two successive episodes that the sensing of incoming waves could be even more complex (see Figure 5e, episode w_1_).

#### Participation of individual agent bees in shimmering

**Figure 9:**
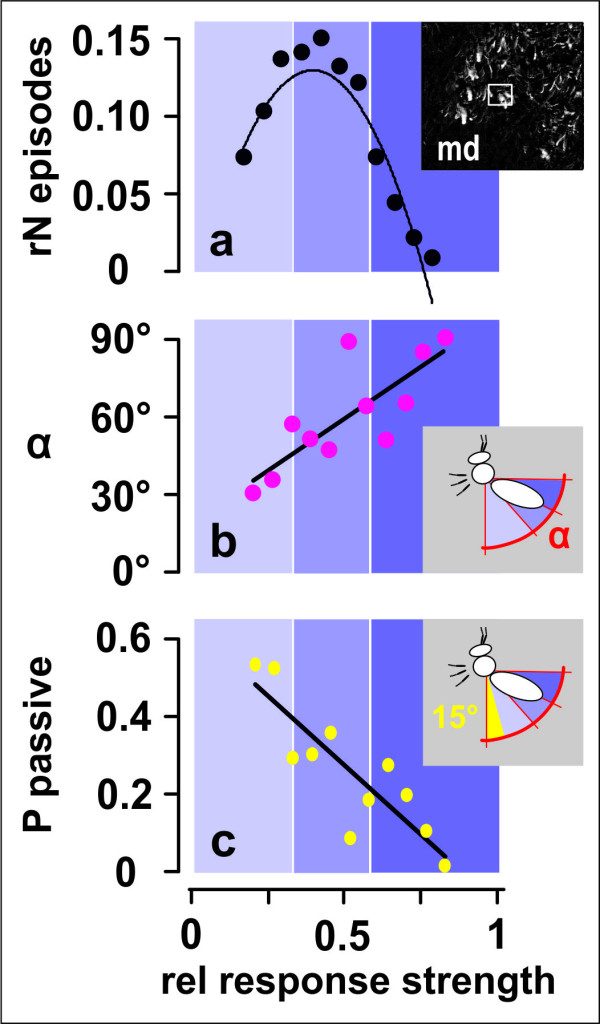
**Correlation between response strength *RS *and flipping angle α**. The response strengths (*RS*) of agent bees assessed by movement detection (see inset: md; Figures 7c-e and 8) were normalized to the maximum value in 605 agents and sorted regarding 10 levels between 0.3 to 0.8 of the relative maximal strength (*rRS*). (a) Gauss distribution of the sorted episodes according to the *rRS *levels (*rN episodes*, relative number of episodes; n_episodes _= 4672). Three *rRS *divisions were defined, marked by different shadings of the violet background. (b) Weighted means of flipping angles in a selected group of episodes (black regression line: y = 78.997x+2.601; n_episodes_sel _= 176; R^2 ^= 0.626; P < 0.01, Spearman Rank Correlation test). (c) The probability of inactivity (no or weak abdominal flipping with flipping angles less than 15°as detected by manual inspection) correlated negatively with the *rRS *(black regression line: y = 0.717x+0.389; n_episodes_sel _= 176; R^2 ^= 0.739; P < 0.001, Spearman Rank Correlation).

Wave episodes were classified into ten response strength (*RS*) levels (Figure 9) regarding the agents' participation in shimmering, but in Figure 8 they were summarized in only three groups, "low", "medium" and" high". The occurrence of shimmering strengths followed a Gaussian distribution (P < 0.05, Kolmogorow-Smirnow test; Figure 9a), which means that the majority of agents participated at medium *RS*. The variation in participation strength can be discerned in *difference *images (see Additional File 4, Movie S4) by their resulting luminance (max Δ*lum*) values. On wave arrival, the motion components (x,y,z: Figure 8a-c) showed initial positive peaks which correlated with *RS*. At the lowest *RS *level (*RS *= 1; not displayed in Figure 8) these transient peaks were not visible, which means that strong positive deflections at higher *RS *levels indicate "active" participation in shimmering. We cross-checked these results with the occurrence of abdominal flipping. In the stereo images (Figure 6d) the abdomens were projected as curved ellipses of decreasing lengths, which allowed assessing the momentary flipping angle. The *RS *values correlated with these angles positively (Figure 9b), and negatively with the occurrence of "passiveness" as defined for Figure 9c whereas flipping angles of 0°-15° represented "passive" contribution. The data confirm that agent-specific *RS *values, and therefore also their correlates, the transient positive positional deflections (Figure 8a-c), are usable measures for the participation in shimmering.

### Applications: Physical Principles of Abdominal Flipping

We also explored the physics of abdominal flipping at the single bee level (Figure 6 and 7, see Additional Files 9, 10 and 11, Movies S6, S7 and S8) and the potential accompanying mechanical cues in colony-intrinsic communication [13]. The thoracic z-motions during shimmering exhibit a prominent initial peak (Figure 8c) due to the abdominal thrusting. This is exemplified for agent 58, where we manually linked the thoracic position and the angular movement of abdominal flipping (Figure 6). Initially (*ff*_rel _= 1-6, defined as *ff *15-20 in Figure 6a), the thorax moved progressively towards the comb by 1 mm in 100 ms, but then (*ff*_rel _= 7-9) it was strongly pushed away from the comb (see Additional File 2, Movie S2 and Additional Files 9, 10 and 11, Movies S6, S7 and S8) with a six-fold greater velocity.

Giant honeybees in the peripheral and outer layers of the bee curtain deposit a lot of watery honey in their abdomens [9], which become much heavier than the counterbalancing heads. Therefore, an abdomen-flipping bee may be simplified, in terms of classic mechanics, as a *torsion pendulum *(see Additional File 11, Movie S8) with an a-centric axis (positioned between thorax and the tarsi of the six legs which fix the thorax to the movable subsurface) and with two disparate masses (the upwards head and the downwards abdomen) on both sides of the rotation centre, oscillating in a (harmonic but damped) curved motion. During abdominal flipping *mass inertia *produces a *reaction force*, which initially presses the agent with the thorax towards the nest, but consecutively, the thrust of the flail-type body with the asymmetry of the pendular masses initiates a *centrifugal *force directed away from the comb. In the collective process of shimmering the centrifugal forces of the surface bees are locally synchronized, which lastly pulls the subsurface layers of the bee curtain concertedly away from the comb (see Additional Files 9, 10 and 11, Movies S6, S7 and S8). Thus, the centrifugal z-component in the second phase of abdominal flipping mainly drives the shimmering process.

## Discussion

### Limits to image acquisition within the given stereoscopic approach

The above application was mainly limited by geometrical and technical constraints. Studying active movements and passive motions at a Giant honeybee nest, such as in shimmering behaviour, needs a thorough 3D analysis with the accuracy of a fraction of a millimetre. We aimed to measure the positional deflections at a resolution of 0.1 mm in all directions of space of hundreds of agent bees simultaneously. This required a stereo baseline of 1 m, which made the stereo system rather large and sometimes difficult to handle under challenging field conditions. To maintain adequate calibration, the cameras were mounted on an aluminium girder that fixed the position of the cameras to each other and enabled movements of the two cameras in a fixed position in front of the bee colony if required. However, we had to compromise the temporal resolution with the illumination conditions at the scene. A nest may be positioned in a dark corner of a veranda or in the bright sun on a tree. Longer exposure times will produce motion blur during fast movements as occur during shimmering. Varying illumination at the nest site was compensated for by locally applied algorithms such as normalized similarity measures. We avoided excessive sunlight, which would prevent reliable measurement by overexposing of nest regions. To satisfy our needs in terms of accuracy and measurement time, two frame-synchronized cameras (see specifications in Additional File 5, Table S1) with a resolution of 4 *Mpx *were used, with fixed lenses (without motorized zoom, focus or iris) to provide calibration stability, and with CMOS image sensors that provide flexible capturing of images of different size at different frame rates (ranging from 60 to more than 500 fps). Lastly, one of the main problems was the enormous data rate produced by the cameras during the experiments. At a frame rate of 60 images with 4 *Mpx *recorded by each camera, an uncompressed data flow of about 480 MB/s had to be managed. Due to the limitation of electric power in the field during our expedition, it was not possible to use hard disks to capture the images in parallel. Therefore, the data had to be stored in the RAM of the computer and written to hard disks later on, constraining acquisition time to RAM storage capacity (in our case: 8 GB RAM, 15 s acquisition time).

### Simultaneous recording of hundreds of agents by stereoscopic imaging

Existing optical tracking methods [36,37] mostly record single views (e.g. in CCTV cameras) of isolated agents in low quantities; if large quantities of agents are viewed, such as hundreds or thousands, motion priors are usually derived from the composite movement of "crowds" [30,37]. These single-view methods are inappropriate for mass phenomena such as shimmering in Giant honeybees, where the precise positional 3D coordinates of individual agents need to be known independently of the global motion. We chose to use 3D stereoscopic imaging, which allows motion analysis of densely packed agents in all directions of space. For this, we developed a system with stabilized tracking performance and resolved ambiguities, which identifies the 3D movements of hundreds of agents simultaneously. So far, shimmering has been documented on traditional film [5,38] in conjunction with classical image analysis, providing only 2D projections of individual bees participating in a 3D process. Laser Doppler vibrometry (LDV) [39] can be useful for some applications, as it also facilitates 3D information. However, it has the disadvantage of delivering data of only one single agent bee over time, and can only pick up transient changes as it is limited by high-pass filter effects.

Here, the stereo tracking method has been modified to measure local, comparably small movements of hundreds of densely packed agents simultaneously, in a flat, but nevertheless three-dimensional stratum. The method is applicable not only to insect clusters such as Giant honeybee nests, but also to processes where positional changes over time have to be monitored on the surface of a stratum of agents with high packing density (such as the growth of plants or cells in 3D, or for displaying deformations of any type of textured surface). For the application of stereoscopic imaging to monitor the dynamics of flocks of birds or fish schools, the methodological approach, in particular the tracking rules, must be adapted.

### Perspectives of analysing shimmering stereoscopically

For the shimmering behaviour of Giant honeybees, the described stereoscopic method produced data that provide behavioural details that could not have been measured by other techniques. It provided evidence for a series of potential mechanoceptive cues perceived by hundreds of surface bees on arrival of a shimmering wave. For example, at the beginning of the experiment, agent 58 drifted slightly away from the comb (< 0.3 mm), starting 300 ms before it strongly participated in the shimmering wave (Figure 5e: episode w_1_, see the positive deflection at *ff *102-115). These "pre-trigger" z-motions of the agents are apparently caused by the abdomen-flipping neighbours. Although small, such passive motions may enable the surface bees to estimate strength and spreading direction of an oncoming wave, and may function to trigger their active participation in shimmering. This response to the wave front is complex: first, the bodies were lifted upwards and pressed towards the comb, and also shifted towards the spreading wave front. Thereafter, the actively shimmering, abdomen-lifting agents pull the subsurface layers massively away from the comb. We propose that the resulting biphasic time course of the z-motion is a subtle colony-intrinsic signal [13,16,35] that provides mechanoceptive information about the momentary defensive state of the colony regarding temporal, spatial and directional patterns. The addressees of such signals are those colony members that are positioned in the subsurface layers and inactive in shimmering, and that are excluded from receiving visual cues.

Hence, 3D analysis of singular aspects of shimmering, such as the thorax positions of surface bees, enhances the understanding of the mechanical basis of abdominal flipping at the single bee level, and of the sensory basis for colony-intrinsic information involved in the spreading of shimmering waves. On the individual bee level, the stereoscopic method enabled a comparison of the mechanics of abdominal flipping with that of a *torsion pendulum*. On the colony level, it allowed an assessment of the mechanic basics of shimmering to explore potential cues for colony-intrinsic communication. In our example, the wave arrived from the right side and drew the agents against the spreading direction. The x-motions of the agents detected can be plausibly explained by the mechanical principle of *shear forces *tangential to the individual agents. This is analogous to wind waves that propagate along the interface between water and air [31-33]; as the wind blows, pressure and friction forces perturb the equilibrium of the water surface, producing waves in which the moving paths of particles near the water surface form circles (regarding monochromatic, linear, and plane waves in deep water [31-33]). Wind waves are therefore a combination of longitudinal (back and forth) and transverse (up and down) motions [23,32,33]. The positive x-motions of agents in horizontally spreading shimmering waves are supposed to correspond to the longitudinal moving of water particles [23,32,33] when directed "backwards".

However, the analogy of shimmering waves with wind waves is limited for at least two reasons. First, wind waves are generated [33] by energy transferred onto the water whereas shimmering is produced by the "active" agents in the bee curtain themselves. Second, in wind waves the restoring force is gravity [33], but in shimmering waves it is the concerted muscular activity of the bees in the layers of the bee curtain acting rectangular to gravity. The positive z-motion in shimmering denotes that abdominal flipping has recruited energy into the previously quiescent parts of the bee curtain, pulling its elastic mass away from the comb with successive damped oscillations (Figure 5e; see Additional File 2, Movie S2 and Additional Files 9, 10 and 11, Movies S6, S7 and S8). This finding questions the analogy to Mexican waves [15] in football stadiums: Although both shimmering and Mexican waves are generated by the energy of the participating agents, the audience in football stadiums does not lift the ground into the air, whereas the wave front during the shimmering process pushes the surface of the bee curtain to the outside direction.

## Conclusions

So far, stereo imaging has been applied to remote sensing [40], close-range photogrammetry [41], material sciences [42,43], medicine [44,45] and tissue mechanics [46]. Although this method can be scaled to a multitude of measurement ranges and corresponding accuracy specifications, to date 3D stereoscopic imaging has not been used for ethological purposes, particularly not for the descriptions of 3D motions of densely packed agents. We propose that it is a valuable tool to study collective behaviours in Giant honeybee colonies, but also, subject to additional adaptations of the method, for swarm behaviours in other insects [47,48], fish [49,50] and birds [51-54] and for special aspects of escape panic in humans [55].

## Competing interests

The authors declare that they have no competing interests.

## Authors' contributions

Conceived and designed the experiments: GK. Performed the experiments: GK TH MM FW. Analyzed the data: GK TH MM FW. Contributed analysis tools: MM GK MR. Wrote the paper: GK MM MR IK. Co-editing: HB. All authors read and approved the final manuscript.

## Supplementary Material

Additional file 1 **Shimmering behaviour in Giant honeybees**. An experimental Giant honeybee nest (140 × 70 cm) attached to a balcony of a hotel in Chitwan, Nepal. On the right, parts of the comb had been removed by a honey hunter some weeks before. With the exception of the mouth zone [8] (bottom right), the bee curtain exhibited a quiescent structure of surface bees, with their heads up and the abdomens down. The movie was recorded with a HD camera with a frame rate of 50 Hz in parallel to the stereo cameras (see Figure 2 for experimental design). At the beginning of the sequence the trigger light (fixed by black adhesive tapes to the wall behind) was turned on for one second. The red spot (middle of the right side of the nest) was produced by the beam of a Laser vibrometer measuring the thoracic z-position of the selected surface bee. Right to the nest, a black-and-white striped dummy wasp was mounted on the cable-car device, and its moving speed and direction were computer-controlled. The dummy wasp provoked shimmering waves before it is seen in the image. Note that the waves originated at the right nest side above the mouth zone. The yellow number on the right bottom gives the time in seconds. The first two waves of this sequence refer to the episodes w_1_, w_2 _of Figure 6 (2.1 MB, MPG). Click here for file

Additional file 2 **Illustration of the triangulation of a selected agent by stereo tracking**. The upper row shows image sequences of the left and right camera regarding the wave episodes w_1 _and w_2 _of agent 58 (for information about cut size and location, see Figure 6 and 7; for further details about z-movement and abdomen flipping, see Figure 7 and 9); the red crosses in the images mark the thorax of agent 58. The bottom bar chart gives the z-values of the thoracic position of agent 58 in mm; positive values refer to directions away from the comb, negative directions towards the comb. The moving red line in the graph marks the momentary time position. Note that the waves in the episodes w_1 _and w_2 _spread from right to left, and that the participation in shimmering of the neighbours of agent 58 varied strongly (1.9 MB, MPG). Click here for file

Additional file 3 **Visualization of the z-movements of all selected agents by stereo tracking during a single wave episode**. Part of the experimental Giant honeybee nest (cf. Additional File 1, Movie S1, Additional File 6, Movie S5 and Additional File 12, Movie S9). The left camera image displays the wave episode w_2 _as shown in Additional File 2, Movie S2 (cf. Figure 6, 7 and 8). Matched and triangulated agents were marked with rounded rectangles showing the identification number. The colours used for coding the z-position refer to 14 steps of towards-comb direction (blue shades from white to dark blue) and off-comb direction (red shades from white to dark red). This film shows the positional information for all 505 agents (cf. Figure 6ab). Note that the waves cause residual motions of the curtain away from the comb for two seconds (as displayed in Figure 6e for agent 58). Numbers refer to frame and time (in ms) information (0.1 MB, MPG). Click here for file

Additional file 4 **Visualization of the differential of z-movements of all selected agents by stereo tracking during a single wave episode**. This film shows the differential of the positional information for all 505 agents (cf. Figure 6c,d,f). For all other details, see legend to Additional File 3, Movie S3 (0.2 MB, MPG). Click here for file

Additional file 5 **Specification of the stereo cameras**. The stereo imaging setup consisted of two global-shutter CMOS cameras, delivering 4 *Mpx *gray-scale images at a frame rate of 60 Hz. The images were recorded and stored by a battery-powered industrial PC. Click here for file

Additional file 6 **Stereo imaging of shimmering**. Detail of the experimental Giant honeybee nest (cf. to Additional File 1, Movie S1). For comparison, the image sequence refers to the same 15 s displaying the shimmering waves shown in Additional File 1, Movie S1. Left and right images of the black-and-white high-speed stereo cameras with frame and time information; images are displayed in inverted luminance values. Note that due to the perspective only the left camera shows the cable car dummy (2.5 MB, MPG). Click here for file

Additional file 7 ***Stereo matching: *the correspondence problem described as a discrete energy minimization task**. Detailed description of the algorithm for *stereo matching *which allows automated identification of corresponding individuals in a pair of stereo images. The problem is challenging because of the inherent similarity of the colony members addressed as agent bees. The problem was formulated as a *discrete energy minimization task*. Click here for file

Additional file 8 ***Stereo matching *by identifying the minimum cut through a reduced graph**. Each segmented bee in the left image was assigned a chain of *M *disparity slots in the right image, according to equation 9. In this example, *M *= 3, one chain corresponds to a column in the graph, and the number of bees in the left image corresponds to the number of columns. Each node contains at the most one bee in the right image, which lies in the respective disparity interval (equation 10). In this example, the red nodes contain a bee, whereas the white nodes are empty. Start and end of each chain are connected from a *source *to a *sink *node, respectively. Links in the graph (black and blue lines) are pairwise connections between nodes, and are assigned capacity values *Ct_0 _(p)*, *Ct (p,i) *and *Cn (p,q,i)*, according to equations 11,13 and 14. Cutting of a t-link is equivalent to selecting the bee above the cut as the correct correspondence. The cut that completely separates *source *from *sink *(a special case is illustrated by the dashed red line) has the smallest sum of cut link capacities; it is called *minimum cut *[28], and results in an optimal assignment of correspondences. Click here for file

Additional file 9 **Illustration of abdominal flipping**. Illustration of abdominal flipping of bees in the neighbourhood of agent 58 (marked by a red full circle) during the wave episode w_2 _(see Figures 6, 7 and 9). Footage in frames and time in ms are displayed (frame rate: 60 Hz). For all other details, see legend to Additional File 3, Movie S3 (0.5 MB, MPG). Click here for file

Additional file 10 **Slow motion of abdominal flipping**. Slow motion of abdominal flipping of bees in the neighbourhood of agent 58 (marked by a red full circle) during the wave episode w_2 _(see Figure 6,7 and 9). Footage in frames and time in ms are displayed (factor slow motion: 10.6). For all other details, see legend to Additional File 12, Movie S9 (2.2 MB, MPG). Click here for file

Additional file 11 **Mechanistic model of an abdomen-flipping Giant honeybee at the surface of a nest**. Model, explaining the movements of the body associated with abdominal flipping according to the y- and z-curves in Figure 9 (not considering the x-movements). The left brown vertical bar denotes the two-sided comb with cells on both sides separated by a mid wall. The two short brown vertical bars between the model bee and the model comb represent the subsurface layers of the bee curtain to which the model bee clings with her six extremities (not shown). In this model, the position of head and thorax, and the distance between thorax and the nearest brown bar was kept constant throughout the flipping process. In the film, the abdominal flipping is simplified: the model bee raises the abdomen by 90°, which provokes y-movements (here displayed in vertical directions) and z-movements (here displayed in horizontal directions). Two phases of the flipping are illustrated. In the initial phase the model bee is pressed towards the subsurface layers, when she actively pulls her body upwards using her extremities, and in the second phase, she moves away from the comb and recovers her initial lower position. In this sketch, the changing interspaces between comb and the subsurface layers additionally illustrate the pressing to and moving away from the comb of the model bee. Numbers indicate frames and time in ms; the sketch slows down real-time abdominal flipping by a factor of 10.78 (0.5 MB, MPG). Click here for file

Additional file 12 **Difference image sequences**. Detail of the experimental Giant honeybee nest (cf. Additional File 1, Movie S1 and Additional File 6, Movie S5). For comparison, the image sequence refers to the same 15 s displaying the shimmering waves shown in Additional File 6, Movie S5. Left, left-camera image; right, difference image giving the subtraction value of Δlum_i,i-1 _= lum_i _- lum_i-1 _between the actual frame *f*_i _and the preceding frame *f*_i-1_; numbers refer to frame and time (in ms) information; the left image shows inverted luminance values (2.5 MB, MPG). Click here for file
