# Supplementary material for: Stereoscopic motion analysis in densely packed clusters: 3D analysis of the shimmering behaviour in Giant honey bees
Source: Front Zool. 2011 Feb 8;8:3. doi: 10.1186/1742-9994-8-3 (PMC3050815; doi:10.1186/1742-9994-8-3)
Supplement: Additional file 5 — Specification of the stereo cameras. The stereo imaging setup consisted of two global-shutter CMOS cameras, delivering 4 Mpx gray-scale images at a frame rate of 60 Hz. The images were recorded and stored by a battery-powered industrial PC. [file 1742-9994-8-3-S5.PDF]

| | |
|---|---|
| Model | DALSA Falcon 4M60 |
| Sensor | CMOS |
| Shutter | Global electronic shutter with exposure control |
| Resolution | 2352 x 1728 |
| Total Data Rate | 320 MHz |
| Max. Frame Rate | 62 fps |
| Pixel Size | 7.4 µm |
| Data Format | 8, 10 bit |
| Data Rate | 4 x 80 MHz |
| Number of Camera Taps | 4 |
| Output | Medium Camera Link |
| Lens Mount | M42 x 1, F |
| Size | 94 x 94 x 50 mm |
| Responsivity | 14 DN/(nJ/cm$^2$) @ 1x gain |
| Dynamic Range | 56 dB |
| Nominal Gain Range | Up to 4x |
| Spectral Responsivity | 400 to 950 nm |
| Size | 94 x 94 x 50 mm |
| Sensor Size | 17,4 x 12,8 mm |
| Mass | <550 g |
| Operating Temp | 0°C to 50°C |
| Power Supply | +12 V to +15 V |
| Power Dissipation | <10 W |
| Regulatory Compliance | CE |
| Control | MDR26 Camera Link |
| Data | Shared with Control |
| Power | Hirose 6 pin |
| Specials | 1000x antiblooming<br>Exposure control<br>Flat field correction (FFC)<br>Exact sensor-alignment |
