# Supplementary material for: Stereoscopic motion analysis in densely packed clusters: 3D analysis of the shimmering behaviour in Giant honey bees
Source: Front Zool. 2011 Feb 8;8:3. doi: 10.1186/1742-9994-8-3 (PMC3050815; doi:10.1186/1742-9994-8-3)
Supplement: Additional file 7 — Stereo matching: the correspondence problem described as a discrete energy minimization task. Detailed description of the algorithm for stereo matching which allows automated identification of corresponding individuals in a pair of stereo images. The problem is challenging because of the inherent similarity of the colony members addressed as agent bees. The problem was formulated as a discrete energy minimization task. [file 1742-9994-8-3-S7.PDF]

Additional file 7 – Text S1. S*tereo matching:* the correspondence problem described as a discrete energy minimization task.

The high level of repetitive similarity hampered the introduction of the maximum similarity criterion that allows a robust identification of true correspondences between agents. Therefore, we formulated the correspondence problem as a discrete energy minimization task (equation 6). The data term $E_{data}(d)$ accumulates the dissimilarity of all assigned correspondences. $E_{smooth}(d)$ is a measure for smoothness, with d defining the disparities between neighbouring bees [22]. The scalar value λ is a user-defined weighting factor. Note that the disparity d in rectified image pairs is the x-offset of corresponding agents, i.e. for a given correspondence $c_n=(p,i)$ as explained in

(Equation 8) $d_{p,i} = x_i^{right} - x_p^{left}$

it is directly proportional to its geometric depth. The goal was to find the set of correspondences c_n for the minimum of $E\,(d)$. For that, a *graph* was constructed using the *minimum-cut / maximum-flow* algorithm [28,29]. A *minimum cut* results in a strong local minimum approximating the global minimum of $E\,(d)$. To construct the graph, we first selected a set of potential correspondences. Every bee in the left image potentially corresponded to every bee in the same row in the right image, with a vertical tolerance of ±6 *px* to account for small segmentation errors.

A similarity term $s_{i,p}$ was assigned to each potential correspondence c_n [22]. This was assessed by the normalized cross-correlation between a segmented region R_i, covering a whole bee, of the agent bee i in the right image, and the corresponding segmented region R_p taken from bee p in the left image. The correlation value was normalized between 0 and 1. For each agent bee in the left image, the number of potential correspondences was reduced by selecting the N most similar candidates and mapping them to the graph by creating a chain of M discrete disparity slots, where each slot represented a disparity interval (d_{min}, d_{max}) according to the

(Equation 9a) $d_{min}\,(m,p) = d_{est}(p) + \left(m - \frac{M}{2}\right) * \frac{2*span}{M}$

(Equation 9b) $d_{max}\,(m,p) = d_{est}(p) + \left(m - \frac{M}{2} + 1\right) * \frac{2*span}{M}$

with $m = 1..M$.

The interval was chosen in a way that at most one correspondence was associated with a given slot defined by

(Equation 10) $\frac{2*span}{M} < \min_{p,q,i}\left(d_{i,p} - d_{i,q}\right)$

The initial disparity d_{est} (p) was estimated by manually establishing four correspondences, which were selected close to the boundaries of the measurement range on the nest. A planar surface was fitted through the supporting points and an approximate disparity was interpolated for each bee in the left image. The graph was then expanded by connecting the chains of M disparity slots containing the N associated possible candidates to the source and sink (Additional File 8, Figure S1). The capacities of the t-links $Ct(p,i)$ that were associated with a possible candidate for correspondence were calculated according to

(Equation 11) $Ct\ (p,i) = 1 - s_{i,p} + K_p$

where K_p was a constant that had to comply with

(Equation 12) $K_p > N*\left|max\left(d_{i,p}\right) - min\left(d_{i,p}\right)\right|$ with $i = 1..N$

with $i = 1..\ N;\ \ d_{i,p}$ was the disparity of the agent bee p to its likely corresponding one i. All other t-links were assigned a capacity $Ct_0(p)$ as described in

(Equation 13) $Ct_0(p) = 2*\left(K_p + 1\right)$

To penalize large disparity differences between neighbouring bees, a smoothness constraint was introduced for the k nearest neighbours. In the graph, the neighbouring constraint is represented by bidirectional n-links that are added to the graph. For two bees p and q the capacity $Cn(p,q,i)$ of the n-links were calculated according to

(Equation 14a) $Cn\ (p,q,i) = K_p*\beta*\left(1 + \frac{1}{\left|d_{i,p} - d_{i,q}\right| + 1}\right)$

if slot i contains a bee for p and q and according to

(Equation 14b) $Cn\ (p,q,i) = K_p*\beta$

if at least a single slot i is empty (with $i = 1...M$, and the scalar parameter β, which was responsible for smoothing the cut). Finally, a cut that separated the source from the sink was

sought, in order to minimize the sum of provoked capacities (Additional File 8, Figure S1). The problem was solved by a *minimum-cut* and *maximum-flow* algorithm [28]. The result was the set of correspondences for the further use in evaluation.

However, the uniqueness constraint could have been violated after optimization. This would happen if more than one bee in the left image was associated with a single bee in the right image. Violations in uniqueness result from an asymmetry in the operation with stereo images was and were resolved in a final step, which (repeated the stereo-matching process backwards for the same paired input images to accept only matches which were successful in both directions.
