# Supplementary figures and images for: Stereoscopic motion analysis in densely packed clusters: 3D analysis of the shimmering behaviour in Giant honey bees
Source: Front Zool. 2011 Feb 8;8:3. doi: 10.1186/1742-9994-8-3 (PMC3050815; doi:10.1186/1742-9994-8-3)

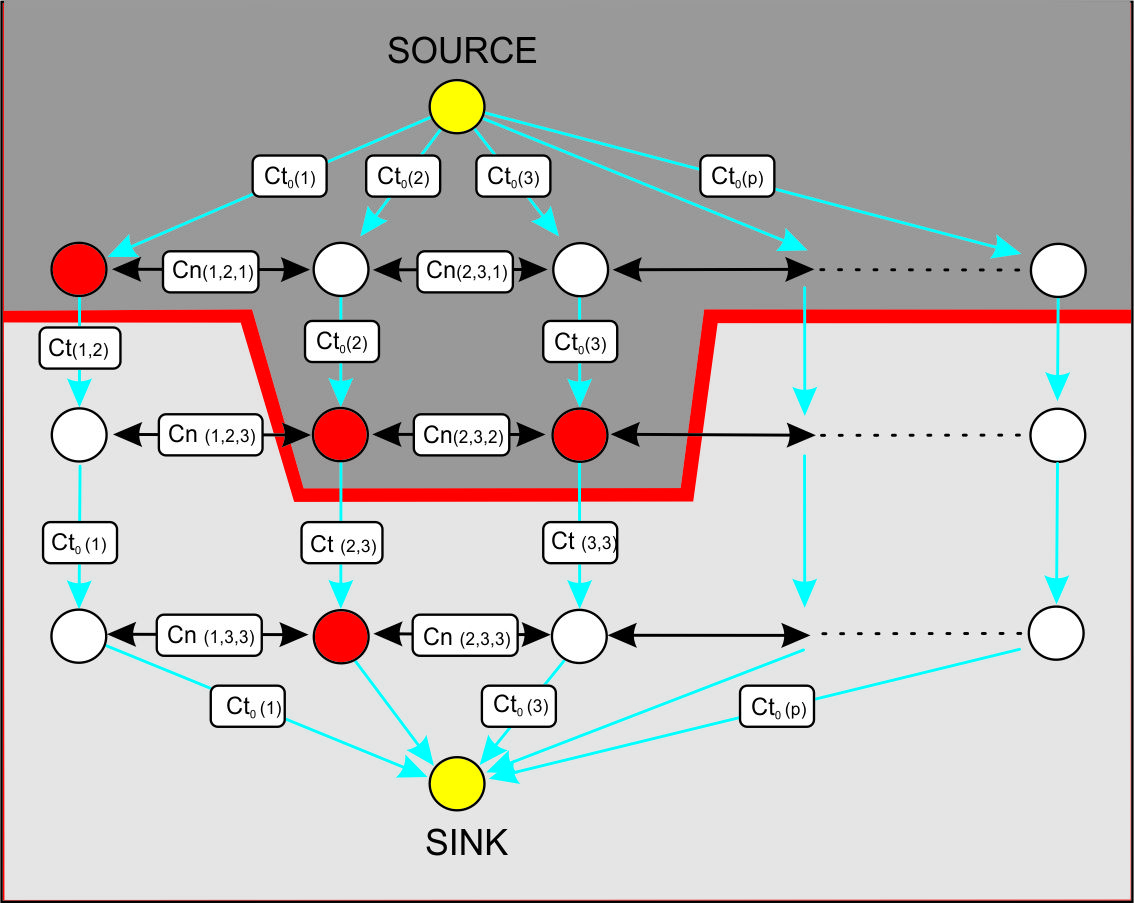

Supplement: Additional file 8 — Stereo matching by identifying the minimum cut through a reduced graph. Each segmented bee in the left image was assigned a chain of M disparity slots in the right image, according to equation 9. In this example, M = 3, one chain corresponds to a column in the graph, and the number of bees in the left image corresponds to the number of columns. Each node contains at the most one bee in the right image, which lies in the respective disparity interval (equation 10). In this example, the red nodes contain a bee, whereas the white nodes are empty. Start and end of each chain are connected from a source to a sink node, respectively. Links in the graph (black and blue lines) are pairwise connections between nodes, and are assigned capacity values Ct0 (p), Ct (p,i) and Cn (p,q,i), according to equations 11,13 and 14. Cutting of a t-link is equivalent to selecting the bee above the cut as the correct correspondence. The cut that completely separates source from sink (a special case is illustrated by the dashed red line) has the smallest sum of cut link capacities; it is called minimum cut [28], and results in an optimal assignment of correspondences. [file 1742-9994-8-3-S8.JPEG]
